# An Emergent Discriminative Learning Is Elicited During Multifrequency Testing

**DOI:** 10.3389/fnins.2019.01244

**Published:** 2019-11-21

**Authors:** Xingui Zhang, Xianhua Ye, Rui Cheng, Qi Li, Zhongju Xiao

**Affiliations:** ^1^Department of Physiology, School of Basic Medical Sciences, Key Laboratory of Psychiatric Disorders of Guangdong Province, Guangdong-Hong Kong-Macao Greater Bay Area Center for Brain Science and Brain-Inspired Intelligence, Key Laboratory of Mental Health of the Ministry of Education, Southern Medical University, Guangzhou, China; ^2^Department of Otolaryngology-Head and Neck Surgery, Nanfang Hospital, Southern Medical University, Guangzhou, China

**Keywords:** medial prefrontal cortex, primary auditory cortex, pure tone, auditory fear conditioning, emergent discriminative learning

## Abstract

In auditory-conditioned fear learning, the freezing response is independent of the sound frequencies used, but the frequency of the conditioned sound is considered distinct from those of unrelated sounds based on electrophysiological responses in the auditory system. Whether an emergent discriminative learning underlies auditory fear conditioning and which nuclei and pathways are involved in it remain unclear. Using behavioral and electrophysiological assays, we found that the response of medial prefrontal cortex (mPFC) neurons to a conditioned auditory stimulus (CS) was enhanced relative to the response to unrelated frequencies (UFs) after auditory fear conditioning, and mice could distinguish the CS during multifrequency testing, a phenomenon called emergent discriminative learning. After silencing the mPFC with muscimol, emergent discriminative learning was blocked. In addition, the pure tone responses of mPFC neurons were inhibited after injection of lidocaine in the ipsilateral primary auditory cortex (A1), and the emergent discriminative learning was blocked by silencing both sides of A1 with muscimol. This study, therefore, provides evidence for an emergent discriminative learning mediated by mPFC and A1 neurons after auditory fear conditioning.

## Introduction

The freezing response in animals induced by fear conditioning is independent of the auditory CS frequency used in the conditioning (Laxmi et al., 2003; Antunes and Moita, 2010; Park et al., 2016; Xiao et al., 2018). After training with single frequency pure tones, using pure tones of different frequencies for testing could cause a similar fear response in animals (Xiao et al., 2018). However, auditory fear conditioning could elicit CS-specific modifications of auditory evoked potentials (AEPs) in A1 (Leon et al., 2017) and best frequency (BF) shifts in the lemniscal auditory system toward the frequency of the conditioned tone (Gao and Suga, 2000; Ji and Suga, 2003, 2007; Suga, 2012). It is unclear whether an emergent discriminative learning that may explain this inconsistency underlies auditory fear conditioning.

Previous studies have demonstrated the involvement of the mPFC in the regulation of conditioned fear learning (Resstel et al., 2006; Almada et al., 2013; Marek et al., 2018; Spalding, 2018). Although mPFC neurons respond to noise (Bubser and Koch, 1994; Kim et al., 2009; Likhtik et al., 2014), clicks (Mihalick et al., 2001; Martin-Cortecero and Nunez, 2016; Janetsian-Fritz et al., 2018) and pure tones (Sierra-Mercado et al., 2006; Fenton et al., 2014; Likhtik et al., 2014), iso-frequency pure tones such as 3 kHz (Fenton et al., 2014), 4 kHz (Sierra-Mercado et al., 2006), and 8 kHz (Likhtik et al., 2014) have been used as CSs in conditioned fear learning. Since there has been no further study on the frequency response properties of mPFC neurons to pure tones, the pure tone responses of mPFC neurons and whether they change after auditory fear conditioning, as well as the connection between the sound responses of mPFC neurons and any hypothetical emergent discriminative learning, remain unclear.

Auditory fear learning and memory are considered to emerge from the integration of conditioned sound stimulation and unconditioned somatosensory stimulation in the amygdala through the auditory and somatosensory pathways, respectively (Romanski and Ledoux, 1993; Rumpel et al., 2005; Han et al., 2007). There are two parallel ascending pathways along the canonical auditory neuraxis: the lemniscal and non-lemniscal pathways (Hu et al., 1994; Jones, 2003). CS information can either directly arrive at the amygdala from the thalamus (low routine) or indirectly via A1 (high routine) (Ledoux, 2000). It is now widely accepted that the specific cortical projection pathway dominates the discrimination between the CS and neutral sounds, while the non-specific thalamic projection pathway plays a leading role in the formation of the fear response (Weinberger, 2007; Antunes and Moita, 2010). Auditory sensory inputs from A1 may arrive at the dorsomedial prefrontal cortex through secondary sensory cortical areas, the insular and temporal cortical areas, then primarily inputs onto the ventromedial prefrontal cortex (Hoover and Vertes, 2007; Denardo et al., 2015; Martin-Cortecero and Nunez, 2016). However, it remains unclear whether the sound input from A1 into mPFC neurons are related to auditory fear conditioning.

In this study, we used auditory fear conditioning and loose-patch recordings, combined with mPFC or A1 injections of muscimol or lidocaine, to answer the questions above. We found that the responses to the CS in mPFC neurons increased relative to those to UFs, and an emergent discriminative learning was elicited whereby the subjects could distinguish between the CS and UFs by the inhibition of fear extinction of the CS during the multifrequency testing after auditory fear conditioning. The emergent discriminative learning was impaired after injections of muscimol into the mPFC. After ipsilateral A1 injections of lidocaine, the responses of mPFC neurons to pure tones disappeared and then recovered. Furthermore, inactivating both sides of A1 with muscimol could also block emergent discriminative learning. These findings provide new insights for further studies of fear discriminative learning in the mPFC.

## Materials and Methods

### Animal Preparation

All procedures were approved by the Animal Care and Use Committee of Southern Medical University, Guangzhou, China. These studies used 172, 6–8 weeks old, female C57BL/6 mice housed under standard colony conditions and maintained on a 12/12 h light/dark cycle. Food and water were supplied *ad libitum*. Every effort was made to minimize the number and suffering of the animals used.

Mice were injected with sodium pentobarbital (60–70 mg/kg, i.p., Sigma, United States) and atropine sulfate (0.25 mg/kg, s.c., Nandao, Hainan, China) for anesthesia and inhibition of respiratory secretions, respectively (Xiong et al., 2013; Xiao et al., 2018). Anesthesia was maintained by supplemental doses (13 mg/kg) of pentobarbital throughout the experimental protocol, ensuring a complete lack of the hind paw withdrawal reflex. The body temperature of the mice was maintained by a heating blanket at 37°C during the surgery. For the electrophysiological experiments, a 1.5 cm long screw clamped into a metal post to fix the head was mounted on top of the exposed skull under a stereotaxic apparatus. An Ag reference electrode was placed under the bone (2.0 mm posterior to bregma, 2.0 mm lateral to midline). For pharmacological manipulation, one 0.5 cm long guide cannula (O.D. 0.41 mm, I.D. 0.25 mm) was embedded in the skull over the mPFC (1.9 mm anterior to bregma, 0.0 mm lateral to midline) or A1 (2.7 mm posterior to bregma, 4.2 mm lateral to midline) with dental cement. A stainless steel obturator (O.D. 0.2 mm) was inserted into the guide cannula to maintain patency. Local anesthetic (lidocaine hydrochloride) and antibiotic ointment (furacin) were applied to the surgical incision after the surgery. Mice were allowed at least 7 days to recover from surgery.

For the electrophysiological experiments, mice were trained to run on a flat plate rotating smoothly around its center on an antivibration table (TMC, Peabody, MA, United States) 1–2 h/day after the recovery period until they learned to stay quiet and run freely in the center of the plate (Abe et al., 2014). One day before electrophysiological experiments, mice were anesthetized with sodium pentobarbital (60–70 mg/kg, i.p.), and a craniotomy (1 mm × 1 mm) was performed over the intended recording region. Then, the dura mater membrane was removed, and the cortex was covered with mineral oil to prevent drying until the recording experiments.

### Sound Stimulation

Pure tones generated using a Tucker-Davis Technologies System 3 (TDT 3, Alachua, FL, United States), amplified using an electrostatic speaker driver (ED1), were delivered through a calibrated free-field speaker (ES1, frequency range 0.5–50 kHz) placed 10 cm in front of the mouse. The pure tone parameters were controlled by BrainWare software through a computer. The free-field speaker was calibrated with 1/8- and 1/4-inch microphones and an amplifier before the experiments (Supplementary Table S1). For the behavioral experiments, pure tones (50 ms duration, 5 ms ramp) of specified frequencies at 80 dB SPL (controlled by a programmable attenuator, PA5) were presented at a rate of 1/s. For the electrophysiological experiments, pure tones of varying frequencies (0.94–30 kHz, in 0.5 octave steps, 50 ms duration, 5 ms ramp) were presented at 80 dB SPL in a randomized sequence at a rate of 1/s.

### Behavioral Procedure

Behavioral experiments consisted of conditional fear training, single frequency testing, multifrequency testing and fear extinction. In this study, we used a delay fear conditioning model to train the subjects. All the processes were video recorded.

In the conditional fear training, mice were trained individually in a single session, 2 min after which a 30 s long CS was activated, and an unconditioned stimulus (US, electric foot shock) was presented during the final 2 s of the CS. In the preliminary experiments, we have trained mice with 4–6 series of paired conditioning, and found that most of the mice had high fear responses with 6 series training when other conditions were fixed. Therefore, all our experiments used six trials, which were separated by a 90 s average (randomly generated from 60 to 120 s, in 5 s steps) intertrial interval (ITI). After the final trial, mice remained in the chambers for an additional 30 s and were then returned to their home cages. The auditory delay fear conditioning was performed in the behavioral chamber (chamber A) (Habitest, Coulbourn Instruments, Holliston, MA, United States). Details of the apparatus have been previously described (Xiao et al., 2018). The conditioning chamber was cleaned with 70% ethanol, and the bedding material was replaced between the sessions.

In the single frequency testing, mice were placed in the testing chamber (chamber B). Details of chamber B were the same as in our previous experiments (Xiao et al., 2018). Mice were tested to pure tones of a single frequency individually. For each test, the mice were put into chamber B for 3 min to habituate and then given 3 min long pure tones without the US. Chamber B was cleaned with 70% ethanol, and the bedding material was replaced between the sessions.

In the multifrequency testing, mice were placed in chamber B. After a 3 min long habituation, a 30 s long CS or an UF without the US was delivered randomly; this step was repeated twelve times, and every pair of presentations was separated by a 30 s average (randomly generated from 20 to 40 s, in 5 s steps) ITI.

In the fear extinction experiment, mice were placed in chamber B. After a 3 min long habituation, 12 trials of a 30 s presentation of the CS or an UF were delivered with 90 s average (randomly generated from 60 to 120 s, in 5 s steps) ITI.

### Loose-Patch Recordings in Awake Mice

Loose-patch recordings were performed on the anti-vibration table in a soundproof room maintained at 24–26°C. Glass pipettes, with a 1 μm tip opening and 5–7 MΩ impedance, were used in all electrophysiological recordings. Artificial cerebrospinal fluid (ACSF; in mM: 124 NaCl, 2.5 KCl, 1.2 NaH_2_PO_4_, 25 NaHCO_3_, 1 MgCl_2_, 2 CaCl_2_, 20 glucose, and 0.5% biocytin, pH 7.2) was used as the intrapipette solution. The pipette capacitance was completely compensated, and the pipette tip was navigated into the recording sites with a micromanipulator (Siskiyou Inc., Grants Pass, OR, United States). Positive pressure (0.5 Psi) was applied to the pipette, which was then advanced until the resistance increased by more than 0.5 MΩ. Then, negative pressure (∼0.5 Psi) was applied to form a 100–250 MΩ seal on the patched neuron, allowing spikes only from the patched cell to be recorded. Signals, filtered with a 300–3,000 Hz bandpass filter and sampled at 20 kHz, were recorded with a MultiClamp 700B amplifier (Axon Instruments/Molecular Devices, Sunnyvale, CA, United States) under voltage-clamp mode, and the command potential was adjusted so that the baseline current was 0 pA (Turner et al., 2005; Xiao et al., 2018). Current spikes were sorted using the BrainWare software (Version 9.21, Tucker-Davis Technologies, Alachua, FL, United States). Spike recordings, stored in DAM and SRC files for off-line analysis, were repeated at least ten times to obtain an array of peristimulus time histograms (PSTH) for each cell. Five-percent sucrose drops were given to the mouse through a tube between the recording sessions.

The recording site in this study was the mPFC, based on the coordinates (1.9 mm anterior to bregma, 0.3 mm lateral to midline) of the Allen Mouse Brain Atlas (Lein et al., 2007), and all neurons recorded were located at a depth of 1500–2500 μm below the pia according to the micromanipulator readings, corresponding to PL and IL. The morphologies and locations of the recorded cells were reconstructed with the standard histological procedure of biocytin staining as in our previous paper (Xiao et al., 2018).

### Inactivation of the mPFC and A1

Muscimol [1 mg/mL, dissolved in physiological saline, Tocris Bioscience, catalog no.0289, United Kingdom, containing 5% biotinylated dextran amines (BDA, Invitrogen, D1828, United States)] and lidocaine, a blocker of voltage-gated Na + channels (20 mg/mL, dissolved in physiological saline, containing 5% BDA) (Martin and Ghez, 1999), were used in the behavioral and electrophysiological experiments, respectively. For the infusions, the obturator in the guide cannula was removed and replaced with an infusion cannula (O.D. 0.21 mm × I.D. 0.11 mm), which penetrated 2.0 or 0.7 mm from the brain surface through the dura mater into the mPFC or A1, respectively, and was connected by a microsyringe (Hamilton 5 μL, Model 7105, Reno, NV, United States). The drugs (100–150 nL in total, gauged with mineral oil) were infused at a rate of 0.2 μL/min using a Hamilton microsyringe controlled by a hydraulic pump (Tehovnik and Sommer, 1997). After injection, the syringe was kept in place for 3 min (Sacco and Sacchetti, 2010). After the experiments, cannula placement and drug infiltration were histologically examined as in our previous experiments (Xiao et al., 2018).

### Statistics and Data Analysis

Data analysis was performed using custom-developed software (MATLAB 2012b, MathWorks). The individuals who performed the analysis were partially blind to the conditions of the experiments, as the data recorded were first pooled together for randomized batch processing. The freezing response provided a measure of conditioned fear. Freezing is defined as the absence of all movements except those related to breathing (Blanchard and Blanchard, 1972) and was quantified during the pure tone presentation. The tone-responsive neuron was identified when overall average response at any time (measured in a time window 0–200 ms after stimulus onset) exceeded the average baseline (measured in a 200 ms time window before stimulus onset) by two SDs. Responses evoked by the same pure tone stimuli were averaged, and the raw response value (measured in a time window 0–200 ms after stimulus onset) was obtained after subtracting the average baseline.

Statistical analysis was computed using SPSS (Version 19, IBM). Before performing appropriate parametric statistics, datasets were first tested for normal distribution (Shapiro-Wilk test) and equal variance (Levene’s test). For two-group comparisons, the unpaired *t*-test or the paired Student’s two-tailed *t*-test – if the two groups were correlated – was performed. For three or more group comparisons, a one-way ANOVA followed by Tukey’s *post hoc* tests was used. For multiple comparisons, in the case of equal variance, the Least-Significant-Difference *post hoc* test was used; otherwise, the Dunnett’s T3 test was used. Data are presented as the mean ± SEM if not otherwise specified. The threshold level of significance was set at *P* < 0.05. Data plotting was carried out using Origin software (version 8, OriginLab), Microsoft PowerPoint software (2016) and Adobe Photoshop software (CS5).

## Results

### Similar Behavioral Responses Evoked by the CS or UFs After Auditory Fear Conditioning

**FIGURE 1:**
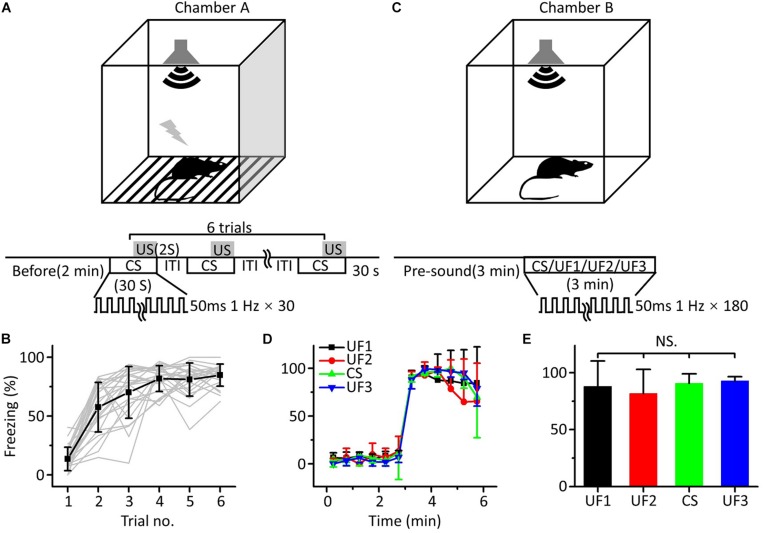
Behavioral responses of auditory fear conditioning in awake mice. **(A)** Training chamber A (upper panel) and training paradigm (lower panel). CS, 30 s long pure tones; US, 2 s long foot shock. **(B)** Freezing responses of awake mice trained with 10.6 kHz pure tones (CS, black square: average; *n* = 24; gray lines: individual responses) to auditory fear conditioning. **(C)** Testing chamber B (upper panel) and single-frequency testing paradigm (lower panel). **(D)** Freezing responses of 1.88 kHz (UF1, black), 3.75 kHz (UF2, red), 10.6 kHz (UF3, green), and 15 kHz-tested (blue) groups were averaged in 30 s windows. **(E)** Freezing responses of the four groups were compared with each other, and the data were the same as in **(D)**. NS, not significant.

To verify that the freezing response induced by auditory fear conditioning was independent of the frequency of the pure tones used as the CS, we used twenty-four mice to perform auditory fear training with 10.6 kHz pure tones (CS). For conditioning, mice were exposed to six pairings of a 30 s long CS and a 2 s long US in chamber A (Figure 1A). The freezing response, quantified as the percentage of time freezing during the presentation of the CS, gradually increased from the first to the sixth trial (Figure 1B). On the following day, mice were randomly divided into four groups. Each group was tested with 3 min-long pure tones of 1.88 (UF1, *n* = 6), 3.75 (UF2, *n* = 6), 10.6 (CS, *n* = 6) or 15 kHz (UF3, *n* = 6) without the US after a 3 min long presound period in chamber B (Figure 1C). The freezing patterns during the 3 min long presound period and the 3 min long pure tone period were averaged over every 30 s (Figure 1D). The freezing patterns for the four groups were not significantly different [Figure 1E, one-way ANOVA, *F*_(__3,20__)_ = 0.542, *P* = 0.659].

It seemed that fear generalization occurred after conditioned fear training, as there was no correlation between the freezing response induced by auditory fear conditioning and the frequency of the pure tones used as the CS, which was consistent with the findings of previous studies (Laxmi et al., 2003; Antunes and Moita, 2010; Park et al., 2016; Xiao et al., 2018).

### Responses of mPFC Neurons to the CS Increased Relative to UFs After Auditory Fear Conditioning

Although the above results showed no differences in the freezing responses between the CS and UFs, some studies have reported that auditory fear conditioning could affect the response of the lemniscal auditory system to the frequency of the conditioned tone (Gao and Suga, 2000; Ji and Suga, 2003, 2007; Suga, 2012). Other papers have shown that the activities of the mPFC were associated with conditioned fear learning (Resstel et al., 2006; Almada et al., 2013; Marek et al., 2018; Spalding, 2018). We thus attempted to study mPFC neurons’ responses to pure tones and whether they changed after auditory fear conditioning.

**FIGURE 2:**
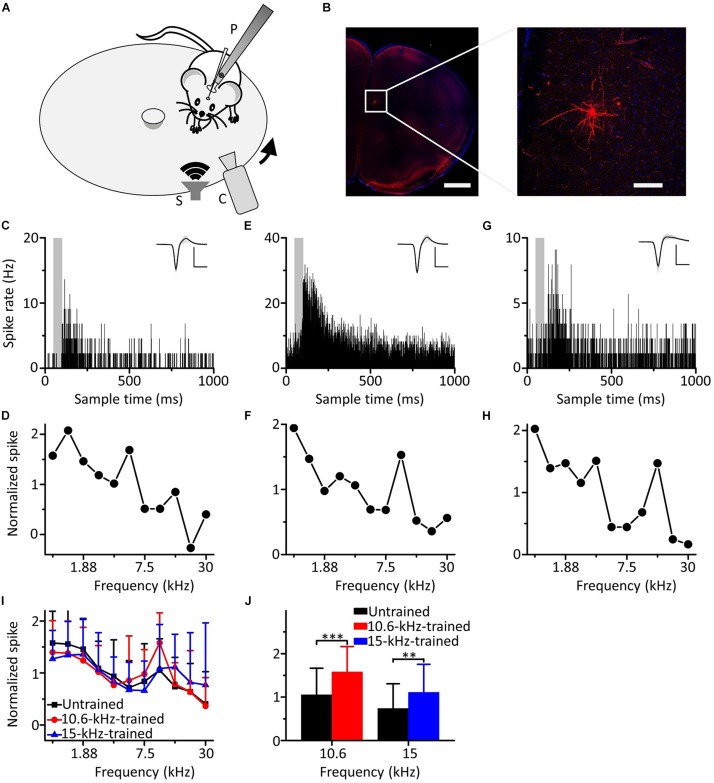
Responses of mPFC neurons to the CS increased relative to those to the UFs after auditory fear conditioning. **(A)** Experimental setup for loose-patch recordings. P, post for fixing animal’s head. S, speaker. C, camera. Arrow indicates rotation of the plate. **(B)** Left panel, confocal image of the mPFC area. Scale: 1,000 μm. Right panel, biocytin-labeled neuron in the mPFC. Scale: 100 μm. **(C)** An example of a mPFC neuron in the untrained group responding to pure tones; the gray area indicates the duration of sound stimulation (50 ms). Inset: average of 20 randomly selected spike waveforms, black line: average, gray lines: individual traces. Scale: 200 pA, 1 ms. **(D)** Normalized spike rate-frequency function for the same cell in **(C)**. **(E,F)** An example of a mPFC neuron in the 10.6 kHz-trained group responding to pure tones. **(G,H)** An example of a mPFC neuron in the 15 kHz trained group responding to pure tones. **(I)** Normalized and averaged spike rate-frequency functions for the untrained group (black), 10.6 kHz trained group (red) and 15 kHz trained group (blue). **(J)** Normalized spike rates to 10.6 kHz pure tones in the untrained group (black) and the 10.6 kHz trained group (red) and to 15 kHz pure tones in the untrained group (black) and the 15 kHz trained group (blue), data are the same as in **(I)**. ^∗∗∗^*P* < 0.001, ^∗∗^*P* < 0.01.

We carried out loose-patch recordings in awake, head-fixed mice on a smoothly rotatable plate (Figure 2A). The coronal sections showed that the neuron was located in the mPFC (Figure 2B, left panel). Morphological characteristics of the neuron are shown in an image obtained under high power microscopy (Figure 2B, right panel). An example mPFC neuron exhibited clear spike responses to pure tones, as manifested by an increased firing rate in the PSTH generated from the responses to the pure tones (Figure 2C). The cell discharged in response to almost all frequencies tested, and the responses to the low frequencies were stronger (Figure 2D). To explore the effects of auditory fear conditioning on the responses of mPFC neurons, we trained mice with CS (either 10.6 or 15 kHz pure tones). The training procedure was the same as in Figure 1A. As shown by the two example mPFC neurons of the mice trained by pairing 10.6 kHz (Figures 2E,F) and 15 kHz pure tones (Figures 2G,H) with the US, the spike responses were clear (Figures 2E,G) and were still stronger to the low frequencies (Figures 2F,H). Most noticeably, clearly increased responses to the CS relative to those to the UFs were observed in both examples (Figures 2F,H). Pure tone-evoked spike responses were observed in approximately 19.7% (44 out of 223 recorded neurons, from 22 mice), 20.2% (38 out of 188 recorded neurons, from 20 mice) and 18.9% (33 out of 175 recorded neurons, from 19 mice) of the total recorded mPFC neurons in untrained, 10.6 and 15 kHz trained mice, respectively. The average of the raw responses for all the frequencies in the untrained and trained groups were shown in Supplementary Table S2, and the raw spike rate-frequency functions was shown in Supplementary Figure S3. Since the data for each group came from different individual mice, we standardized the average response of each neuron and then aggregated all the data for analysis to reduce individual differences. There was a similar trend in all three groups in which the responses to the low frequencies were stronger, as shown by the normalized average spike rate-frequency functions (Figure 2I). On the other hand, the responses to the CS relative to those to the UFs of mPFC neurons in both the 10.6 and 15 kHz-trained mice were significantly stronger than those in untrained mice (Figure 2J, unpaired two-tailed *t*-test, 10.6 kHz trained vs. untrained: *t* = 3.969, *df* = 80, *P* < 0.001; 15 kHz trained vs. untrained: *t* = 2.707, *df* = 75, *P* = 0.008). The normalized responses to all frequencies in untrained and trained conditions were shown in Supplementary Table S3. When we performed additional experiments with the CS at a low frequency of 1.88 kHz (Supplementary Figure S4), similar result was obtained.

Therefore, our results showed that the responses of the neurons in the mPFC to the CS increased relative to those to the UFs after auditory fear conditioning.

### Different Behavioral Responses Evoked Between the CS and UFs After Auditory Fear Conditioning

Since the response of the neurons in the mPFC was not consistent with the behavioral response after auditory fear conditioning, we speculated that the different results may be attributed to the different conditions of sound stimulation; while pure tones of a single frequency were used in the behavioral testing, the electrophysiological recordings were performed with pure tones of multiple frequencies.

**FIGURE 3:**
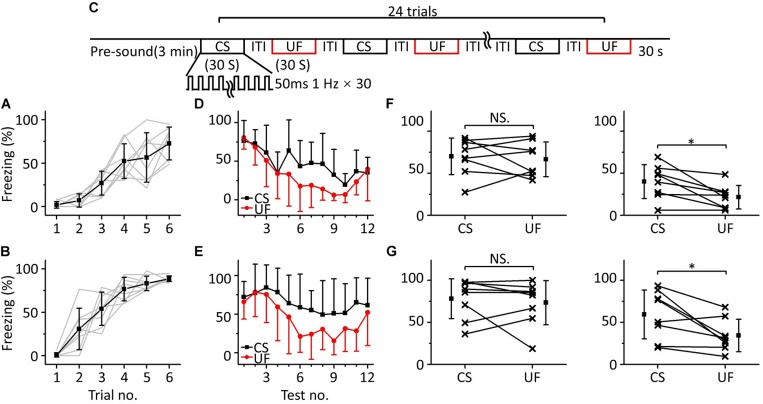
Two-frequency testing after auditory fear conditioning. **(A,B)** Freezing responses of awake mice trained with 3.75 kHz (**A**; *n* = 8) or 15 kHz (**B**; *n* = 8) pure tone CSs. **(C)** Two-frequency testing paradigm. **(D)** Freezing responses of 3.75 kHz (CS, black) and 15 kHz (UF, red) pure tones in the 3.75 kHz trained group. **(E)** Freezing responses of 15 kHz (CS, black) and 3.75 kHz (UF, red) pure tones in the 15 kHz trained group. **(F)** Average freezing responses in the 3.75 kHz trained group. Left panel, average freezing responses of the former part. Right panel, average freezing responses of the latter part. **(G)** Average freezing responses in the 15 kHz trained group. NS, not significant. ^∗^*P* < 0.05.

To test this hypothesis, we randomly divided sixteen mice into two groups for training with 3.75 kHz (Figure 3A, *n* = 8) or 15 kHz pure tones (Figure 3B, *n* = 8) but tested with a CS and an UF (3.75 and 15 kHz pure tones, respectively, for the 3.75 kHz group, and the opposite for the 15 kHz group) in the same testing session on the second day (Figure 3C). To distinguish these testing procedures from that shown in Figure 1C, we refer to them as multifrequency testing and single-frequency testing, respectively. At the beginning of the multifrequency testing, the freezing responses induced by the CS and the UF were similar in both groups but seemed to be different at the end (Figures 3D,E). Then, we divided the multifrequency testing into “former” and “latter” parts, the first three and the last nine presentations of the 30 s CS and the 30 s UF, respectively. The former part showed no difference in the freezing responses between the CS and the UF either in the 3.75 kHz (Figure 3F, left panel, paired *t*-test, *t* = 0.461, *df* = 7, *P* = 0.659) or in the 15 kHz trained group (Figure 3G, left panel, paired *t*-test, *t* = 0.589, *df* = 7, *P* = 0.575). Nevertheless, the freezing response to the CS was significantly higher than that to the UF in the latter part in the 3.75 kHz-trained group (Figure 3F, right panel, paired *t*-test, *t* = 2.959, *df* = 7, *P* = 0.021), as well as in the 15 kHz trained group (Figure 3G, right panel, paired *t*-test, *t* = 2.876, *df* = 7, *P* = 0.024).

**FIGURE 4:**
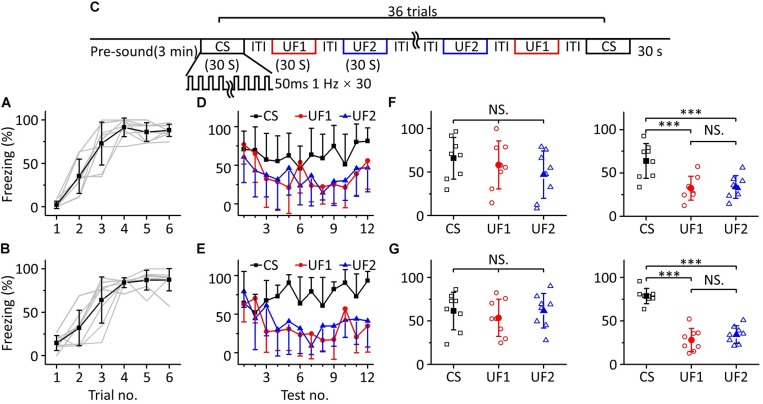
Three-frequency testing after auditory fear conditioning. **(A,B)** Freezing responses of awake mice trained with 10.6 kHz (**A**; *n* = 8) or 3.75 kHz (**B**; *n* = 8) pure tone CSs. **(C)** Three-frequency testing paradigm. **(D)** Freezing responses of 10.6 kHz (CS, black), 3.75 kHz (UF1, red), and 1.88 kHz (UF2, blue) pure tones in the 10.6 kHz trained group. **(E)** Freezing responses of 3.75 kHz (CS, black), 10.6 kHz (UF1, red) and 1.88 kHz (UF2, blue) pure tones in the 3.75 kHz trained group. **(F)** Average freezing responses in the 10.6 kHz trained group. Left panel, average freezing responses of 10.6 kHz (CS, black), 3.75 kHz (UF1, red) and 1.88 kHz (UF2, blue) pure tones in the former part. Right panel, average freezing responses in the latter part. **(G)** Average freezing responses in the 3.75 kHz trained group. NS, not significant. ^∗∗∗^*P* < 0.001.

To confirm this result, we devised other behavioral experiments, in which we randomly divided sixteen mice into two groups for training with 10.6 kHz (Figure 4A, *n* = 8) or 3.75 kHz pure tones (Figure 4B, *n* = 8), but the frequencies of pure tones in the multifrequency testing were 10.6 kHz (CS), 3.75 kHz (UF1), and 1.88 kHz (UF2) or 3.75 kHz (CS), 10.6 kHz (UF1), and 1.88 kHz (UF2), respectively (Figure 4C). A similar result was observed as in the experiment shown in Figure 3; that is, in the early stage of the multifrequency testing, the freezing responses induced by the CS, UF1, and UF2 were similar but were different in the latter stage (Figures 4D,E). There was no significant difference in the freezing responses induced by the CS, UF1, and UF2 in the former part either in the 10.6 kHz trained group [Figure 4F, left panel, one-way ANOVA, *F*_(2, 21)_ = 1.027, *P* = 0.375. Least-Significant-Difference test, CS vs. UF1: *P* = 0.566, CS vs. UF2: *P* = 0.169, UF1 vs. UF2: *P* = 0.409] or in the 3.75 kHz trained group [Figure 4G, left panel, one-way ANOVA, *F*_(2, 21)_ = 0.387, *P* = 0.684. Least-Significant-Difference test, CS vs. UF1: *P* = 0.464, CS vs. UF2: *P* = 0.975, UF1 vs. UF2: *P* = 0.446]. In the latter part, the freezing response induced by the CS was significantly higher than that induced by the UF1 and that induced by the UF2 in the 10.6 kHz-trained group [Figure 4F, right panel, one-way ANOVA, *F*_(2, 21)_ = 9.86, *P* < 0.001. Least-Significant-Difference test, CS vs. UF1: *P* < 0.001, CS vs. UF2: *P* < 0.001] and in the 3.75 kHz trained group [Figure 4G, right panel, one-way ANOVA, *F*_(2, 21)_ = 51.192, *P* < 0.001. Least-Significant-Difference test, CS vs. UF1: *P* < 0.001, CS vs. UF2: *P* < 0.001]. Furthermore, there was no significant difference in the freezing responses to the UF1 and the UF2 (10.6 kHz-trained group: *P* = 0.857, 3.75 kHz trained group: *P* = 0.275).

The results above indicate that the mice could distinguish between the CS and UFs after auditory fear conditioning when they were exposed to pure tones of multiple frequencies for long enough, that is, an emergent discriminative learning was elicited due to additional multifrequency testing rather than just conditioned fear training.

### Fear Extinction of the CS Was Inhibited in MultiFrequency Testing

**FIGURE 5:**
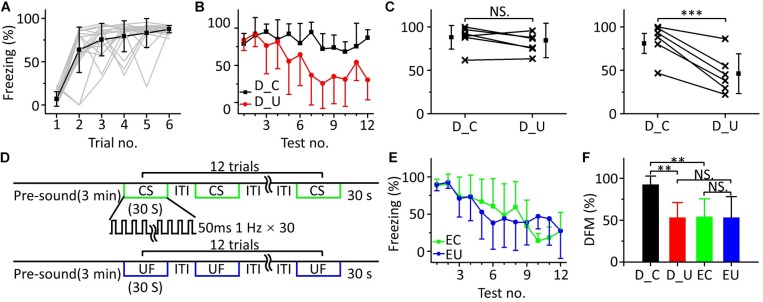
Fear extinction of CS was inhibited in multifrequency testing. **(A)** Freezing responses of awake mice trained with a 10.6 kHz pure tone CS (*n* = 18). **(B)** Freezing responses of D_C (black) and D_U (red). **(C)** Average freezing responses in the discrimination group. **(D)** Fear extinction paradigm. Fear extinction included a 3 min presound period and 12 repetitions of 30 s exposures to the CS (upper panel) or UF (lower panel). **(E)** Freezing responses of EC (green) and EU (blue). **(F)** The DFMs of D_C (black), D_U (red), EC (green), and EU (blue). D_C, discrimination group tested with the CS. D_U, discrimination group tested with the UF. CS, 10.6 kHz pure tones. UF, 3.75 kHz pure tones. EC, CS-extinction group. EU, UF-extinction group. DFM, degree of fear maintenance. NS, not significant. ^∗∗∗^*P* < 0.001, ^∗∗^*P* < 0.01.

In the above multifrequency testing, it seemed that fear extinction of the CS was somewhat inhibited, leading to distinguishing the CS from the UFs. To test whether there was indeed inhibition resulting in emergent discriminative learning, we trained eighteen mice with 10.6 kHz pure tones (Figure 5A) and randomly divided them into three groups: a discrimination group (*n* = 6), a CS (10.6 kHz pure tone)-extinction group (EC, *n* = 6) and an UF (3.75 kHz pure tone)-extinction group (EU, *n* = 6) for testing the next day. Mice in the discrimination group were tested with the CS and UF under the same experimental procedure as in Figure 3C. The freezing responses induced by the CS and UF were similar in the early stage of the multifrequency testing but were different in the latter stage (Figure 5B). In the former part of the multifrequency testing, there was no significant difference in the freezing responses induced by the CS and UF (Figure 5C, left panel, paired *t*-test, *t* = 0.925, *df* = 5, *P* = 0.397). The freezing response induced by the CS was obviously larger in the latter part (Figure 5C, right panel, paired *t*-test, *t* = 6.392, *df* = 5, *P* < 0.001). The EC and EU were exposed to CS (Figure 5D, upper panel) or UF (Figure 5D, lower panel), respectively, to perform fear extinction. Extinction of conditioned fear was evident in both groups (Figure 5E). To quantify the extinction of conditioned fear, we defined the ratio of the average value of the freezing response in the latter part to that in the former part as the degree of fear maintenance (DFM). The greater the DFM, the smaller the extinction of conditioned fear. The results showed that there were significant differences in the DFM among mice of the discrimination group tested with the CS (D_C), the discrimination group tested with the UF (D_U), the EC and the EU [Figure 5F, one-way ANOVA, *F*_(__3,20__)_ = 6.116, *P* = 0.004]. The DFM in the D_C was significantly higher than that in the D_U (Least-Significant-Difference test, *P* = 0.002) and was also significantly higher than that in the EC (P = 0.003). The DFMs in the D_U and in the EC were no different from that in the EU (D_U vs. EU: *P* = 0.988, EC vs. EU: *P* = 0.921).

Thus, the emergent discriminative learning resulting from the inhibition of fear extinction of the CS may be affected by the interaction between the CS and the UF rather than by their original different extinctions of conditioned fear. On the other hand, we observed that the discrimination between the CS response and the UF response seemed to decrease in the last three trials. Therefore, we devised a supplementary behavioral experiment in which we extended the series of trials from 12 to 24: the discrimination between the CS response and the UF response decreased in the latter stage (Supplementary Figure S1). It is easy to understand that the freezing response will gradually decrease when mice are exposed to sounds without an US for long enough, resulting in the discrimination score decreasing as well.

### Inactivating the mPFC Blocked Emergent Discriminative Learning

We observed a consistency in the results between the electrophysiological recordings of mPFC neurons and animal behavior in the multifrequency testing after auditory fear conditioning. Previous studies have revealed that the mPFC exerts a modulatory effect on the expression and extinction of fear conditioning related to discrimination learning (Vidal-Gonzalez et al., 2006; Sierra-Mercado et al., 2011; Albrechet-Souza et al., 2013; Fenton et al., 2014).

**FIGURE 6:**
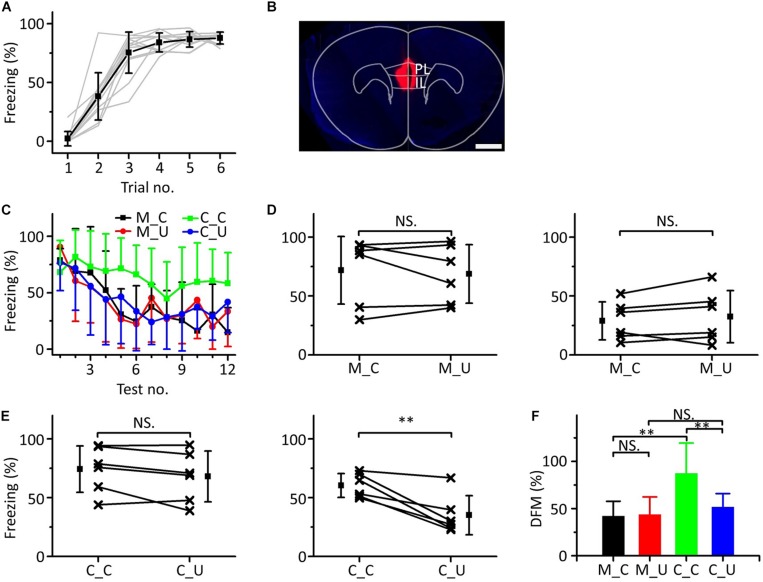
Effects of inactivation of the mPFC on emergent discriminative learning. **(A)** Freezing responses of awake mice trained with a 10.6 kHz pure tone CS (*n* = 12). **(B)** The location of inactivated mPFC in a frontal section, indicated by BDA fluorescence. Scale bar, 1000 μm. **(C)** Freezing responses to the CS (black), UF (red) in the muscimol group and the CS (green), UF (blue) in the control group. **(D)** Average freezing responses in the muscimol group. **(E)** Average freezing responses in the control group. **(F)** The DFMs of M_C (black), M_U (red), C_C (green) and C_U (blue). CS, 10.6 kHz pure tones. UF, 3.75 kHz pure tones. M_C, muscimol group tested with CS. M_U, muscimol group tested with UF. C_C, control group tested with CS. C_U, control group tested with UF. NS, not significant. ^∗∗^*P* < 0.01.

To determine whether the mPFC was related to emergent discriminative learning, we silenced the mPFC with muscimol to observe whether the behavior of mice changed in the multifrequency testing after auditory fear conditioning. On the first day of the behavioral experiment, a 10.6 kHz pure tone was used as the CS to train mice (Figure 6A). The next day, the mice were randomly divided into two groups: the muscimol group (*n* = 6) and the control group (*n* = 6). Approximately 15 min before the multifrequency testing, the mice in the muscimol group were injected with 100–150 nL muscimol. The location of the buried tube injection was determined from the BDA-processed tissue (Figure 6B). The control group mice were given the same dose of saline. Then, the multifrequency tests were performed with 10.6 kHz (CS) and 3.75 kHz pure tones (UF) in both groups, as shown in Figure 3C. The mice in the muscimol group showed obvious extinction of conditioned fear, and the freezing responses induced by the CS and UF were similar over the entirety of the multifrequency testing. The results of the control group were similar to those shown in Figures 3D,E (Figure 6C). In the muscimol group, there was no significant difference in the freezing responses induced by the CS and UF both in the former part (Figure 6D, left panel, paired *t*-test, *t* = 0.566, *df* = 5, *P* = 0.596) and in the latter part (Figure 6D, right panel, paired *t*-test, *t* = 1.086, *df* = 5, *P* = 0.327). In the control group, there was no significant difference between the freezing responses induced by the CS and the UF in the former part (Figure 6E, left panel, paired *t*-test, *t* = 1.826, *df* = 5, *P* = 0.127). However, the freezing response caused by the CS was significantly greater in the latter part (Figure 6E, right panel, paired *t*-test, *t* = 4.417, *df* = 5, *P* = 0.007). After comparing the DFMs between the two groups, we found that there were significant differences in the DFMs between the muscimol group and the control group caused by the CS or the UF [Figure 6F, one-way ANOVA, *F*_(__3,20__)_ = 0.861, *P* = 0.005]. Among them, the DFMs of the muscimol group tested with the CS (M_C) and that of the control group tested with the UF (C_U) were significantly lower than that of the control group tested with the CS (C_C) (Least-Significant-Difference test, M_C vs. C_C: *P* = 0.002, C_U vs. C_C: *P* = 0.009). There were no significant differences in the DFMs between the M_C and muscimol groups tested with the UF (M_U) (*P* = 0.905) or between the M_U and the C_U groups (*P* = 0.521).

The results showed that by inactivating the mPFC with muscimol, the emergent discriminative learning of mice after auditory fear conditioning was blocked; that is, the mPFC participated in emergent discriminative learning.

### Inactivating Ipsilateral A1 Affects the Pure Tone Response of the mPFC

**FIGURE 7:**
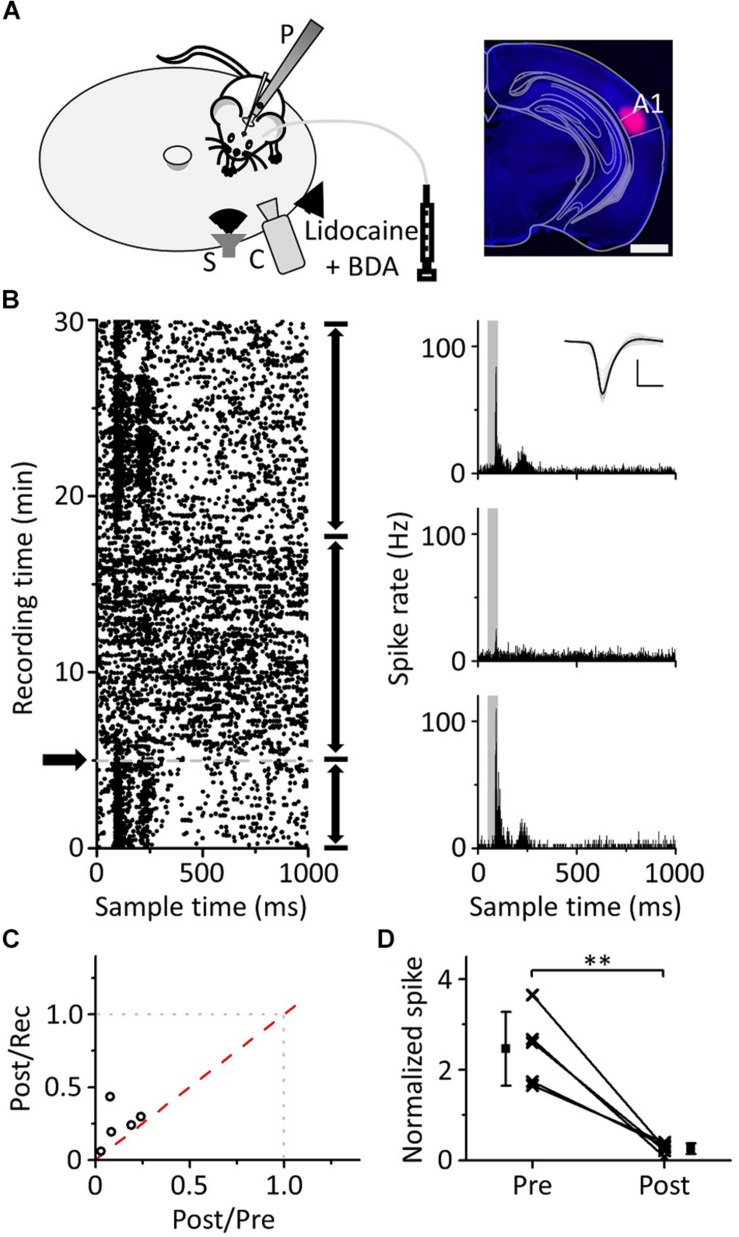
Effects of inactivation of the ipsilateral A1 on the responses of the mPFC to pure tones. **(A)** Left panel, diagram of the recording and lidocaine (coapplied with BDA) injection systems. Right panel, confocal image of the A1 area. Scale: 1,000 μm. **(B)** The responses (left panel, raster plots; right panel, PSTHs) of the neuron in the mPFC before and after lidocaine injection (arrows) to pure tones. Inset: spike waveforms. Scale: 200 pA, 1 ms. **(C)** The spike rate ratio (*n* = 5) of Post/Rec vs. that of Post/Pre. **(D)** The normalized responses of neurons in the mPFC before and after lidocaine injection. The responses are normalized by the average spike rate of the sound response period during the recording process, and the data are the same as in **(C)**. ^∗∗^*P* < 0.01.

Previous literature has indicated that auditory information ascending to the mPFC mainly comes from A1; when using lidocaine to silence A1, auditory-evoked potentials (AEPs) induced by clicks were significantly reduced both in A1 and the mPFC (Martin-Cortecero and Nunez, 2016). Hoover and Vertes used retrograde tracing techniques and found that the mPFC received afferent projections from the ipsilateral auditory cortex rather than the contralateral auditory cortex (Hoover and Vertes, 2007). To confirm that the pure tone response of mPFC neurons originated from A1, we used lidocaine to inactivate the ipsilateral A1 while performing loose-patch recordings of mPFC neurons (Figure 7A, left panel) to observe whether the pure tone response changed. The site of the buried tube injection was determined from the BDA-processed tissue (Figure 7A, right panel). The scatter plot of the example neuron’s response to pure tones (15 kHz) was extracted, and the ipsilateral A1 was inactivated during the recording process (Figure 7B, left panel). PSTHs before and after inactivation were plotted (Figure 7B, right panel). The response of mPFC neurons to pure tones decreased significantly after ipsilateral A1 inactivation, then subsequently recovered. Five mPFC neurons were recorded with a single frequency, evoking a clear response (Figure 7C). We found that the pure tone response of mPFC neurons was significantly inhibited after inactivation of A1 (Figure 7D, paired *t*-test, *t* = 5.625, *df* = 4, *P* = 0.005). The results showed that the pure tone response of mPFC neurons was mainly derived from A1.

### Inactivating Both Sides of A1 Blocked Emergent Discriminative Learning

**FIGURE 8:**
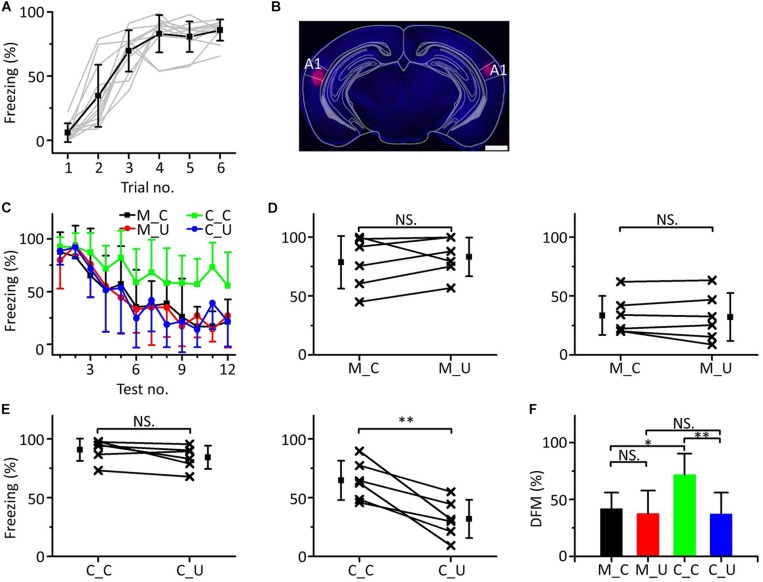
Effects of inactivation of both sides of A1 on emergent discriminative learning. **(A)** Freezing responses of awake mice trained with a 10.6 kHz pure tone CS (*n* = 12). **(B)** The location of inactivated A1 in a frontal section, indicated by BDA fluorescence. Scale bar, 1000 μm. **(C)** Freezing responses to the CS (black), UF (red) in the muscimol group and the CS (green), UF (blue) in the control group. **(D)** Average freezing responses in the muscimol group. **(E)** Average freezing responses in the control group. **(F)** The DFMs of M_C (black), M_U (red), C_C (green), and C_U (blue). CS, 10.6 kHz pure tones. UF, 3.75 kHz pure tones. NS, not significant. ^∗^*P* < 0.05, ^∗∗^*P* < 0.01.

Since the mPFC participates in emergent discriminative learning and its pure tone information mainly comes from A1, we silenced A1 bilaterally to observe whether A1 was involved in the emergent discriminative learning. We used a 10.6 kHz pure tone as the CS to train mice (Figure 8A). Twenty-four h after training, the mice were randomly divided into the muscimol group (*n* = 6) and the control group (*n* = 6). Approximately 15 min before the testing, mice in the muscimol group were injected with 100–150 nL muscimol in both sides of A1. The location of the buried tube injection was determined from the BDA-processed tissue (Figure 8B). The mice in the control group were given the same dose of saline. Then, the multifrequency tests were carried out with the CS (10.6 kHz pure tones) and an UF (3.75 kHz pure tones) in both groups, as shown in Figure 3C. The results were similar to those with the mPFC injections (Figure 8C). In the muscimol group, there was no significant difference in the freezing responses induced by the CS and UF in the former part (Figure 8D, left panel, paired *t*-test, *t* = 0.860, *df* = 5, *P* = 0.429) and the latter part (Figure 8D, right panel, paired *t*-test, *t* = 0.569, *df* = 5, *P* = 0.594). In the control group, there was no significant difference in the freezing responses between the CS and the UF in the former part (Figure 8E, left panel, paired *t*-test, *t* = 2.071, *df* = 5, *P* = 0.093), but in the latter part, the freezing response caused by CS was significantly greater (Figure 8E, right panel, paired *t*-test, *t* = 4.430, *df* = 5, *P* = 0.007). Furthermore, there were significant differences in the DFMs among the M_C, M_U, C_C, and C_U [Figure 8F, one-way ANOVA, *F*_(3, 20)_ = 5.064, *P* = 0.009]. The DFMs of the M_C and C_U were lower than that of the C_C (Least-Significant-Difference test, M_C vs. C_C: *P* = 0.010, C_U vs. C_C: *P* = 0.003). There were no significant differences in the DFMs between the M_C and M_U (*P* = 0.678) or between the M_U and C_U (*P* = 0.981).

Accordingly, the result of inactivation of both sides of A1 by muscimol was similar to that of inactivation of the mPFC, in which the emergent discriminative learning after auditory fear conditioning was blocked, which further confirmed that the pure tone information ascending to the mPFC and participating in the emergent discriminative learning after auditory fear conditioning may mainly come from A1.

## Discussion

In this study, we explored the pure tone response properties of mPFC neurons before and after auditory fear conditioning, the emergent discriminative learning elicited, the role of mPFC in the learning after auditory fear conditioning, and its pathway of activation by combining behavioral, electrophysiological and pharmacological assays. We observed four major findings. (1) Some neurons of the mPFC had broadband responses to pure tones and stronger responses to low frequencies, and their responses to the CS were enhanced relative to those to UFs after auditory fear conditioning. (2) Multifrequency testing after conditioned fear training could gradually reveal emergent discriminative learning. (3) The mPFC participates in emergent discriminative learning. (4) Pure tone information ascending to mPFC neurons, which mediates the emergent discriminative learning, mainly comes from A1.

In the preliminary experiments, we trained mice at lower intensities (i.e., 60 dB, 10.6 kHz), and found that many mice had low and/or unstable fear responses when other conditions were fixed (presumably the high intensities are more effective for training). Therefore, we used a 10.6 kHz pure tone at 80 dB as the CS for auditory fear conditioning in mice (Figures 1A,B) and tested them with different frequencies of pure tones (UF1: 1.88 kHz, UF2: 3.75 kHz, CS: 10.6 kHz or UF3: 15 kHz) on the second day (Figure 1C). The results showed that pure tones of different frequencies could cause similar fear responses in animals (Figures 1D,E). This result agreed with our previous paper (Xiao et al., 2018) and was also supported by other literature. For example, in one study the animal acquired fear of a 10 kHz pure tone and generalized it to a 2 kHz pure tone after auditory fear conditioning (Antunes and Moita, 2010). A recent study showed that synaptic plasticity in the amygdala differed under different auditory CSs conditions, but there were no significant differences in the freezing responses (Park et al., 2016). Thus, in the single-frequency testing after auditory fear conditioning, animals may have generalized not only the fear response to the CS but also to the UFs.

We performed loose-patch recordings of mPFC neurons to observe their pure tone responses and changes in those responses affected by conditioned fear training (Figure 2). Our results showed that some mPFC neurons in awake mice responded to pure tones (Figures 2C–H), which was consistent with previous reports (Sierra-Mercado et al., 2006; Fenton et al., 2014; Likhtik et al., 2014). There was little significant difference in the percentage of mPFC neurons responding to pure tones with and without auditory fear conditioning. Previous studies have mentioned that the activity of mPFC neurons increased during fear learning, fear consolidation or fear retrieval (Popa et al., 2010; Fenton et al., 2014; Likhtik et al., 2014). However, these studies used the field potential recording method (Popa et al., 2010; Fenton et al., 2014; Likhtik et al., 2014) or multiunit recording method (Popa et al., 2010; Fenton et al., 2014), instead of looking at the number of individual neurons responding to pure tones in the mPFC and how their responses changed as a result of auditory fear conditioning. Instead, we recorded the response of a single neuron to pure tones using loose-patch recordings. Frankly, our results are limited to our experimental conditions. We are not sure whether this percentage can be increased with training for longer period of time. Fenton and colleagues suggest that a similar increase in LFP activity occurred after learning as is observed for unit activity in PL and IL (Fenton et al., 2014). Maybe the neurons in mPFC respond to pure tones are specific, and the training just changes some of the characteristics of their responses after task-training that does not increase the percentage. There was no contradiction between the results of our study and those of the above studies. Although single-cell recording of mPFC neurons has been performed previously in a fear experiment, the cue used was olfactory stimulation (Tan et al., 2011).

In preliminary experiments, we found that most tone-responsive neurons in mPFC had high thresholds for pure tones (70–80 dB). Although mice respond to tones of much lower intensities in fear conditioning paradigms (Heffner et al., 1994; Heffner et al., 2001; Froemke et al., 2013), we have to use higher intensity tones to record electrophysiological responses of mPFC neurons. In this study, we used pure tones of only one intensity and eleven frequencies (80 dB SPL, 0.94–30 kHz, in 0.5 octave steps) to increase the number of sound stimuli because of the strong spontaneous response of the mPFC in awake mice (Popa et al., 2010; Fenton et al., 2014); the signal-to-noise ratio of the response was then enhanced. We found that individual mPFC neurons lacked the specific response properties of classical auditory nuclei, such as narrow frequency tuning curves and an obvious distribution of topological structure, but did possess a wide range of frequency responses to pure tones (Figures 2D,F,H). mPFC neurons had a stronger response to pure tones of low frequencies (below 15 kHz) but a weaker response to pure tones of high frequencies (above 15 kHz) with or without auditory fear conditioning, which coincided with previous studies on the mPFC using a low-frequency pure tone as a cue (Sierra-Mercado et al., 2006; Fenton et al., 2014; Likhtik et al., 2014). In addition, our study showed that the response to the CS was enhanced relative to that to the UFs after auditory fear conditioning (Figures 2I,J), which was also in line with the description of the selective increase of the response of mPFC neurons to the CS after fear learning in previous studies (Fenton et al., 2014; Likhtik et al., 2014).

To confirm the cause of the inconsistency between the response of the mPFC and the behavioral response after auditory fear conditioning, multifrequency testing was performed (Figures 3, 4). We found that in the early stage of the testing, animals could not distinguish the CS from the UF (Figure 3F, left panel, Figure 3G, left panel, Figure 4F, left panel, Figure 4G, left panel), which was consistent with the results of the above behavioral experiments and the descriptions in prior literature about fear generalization (Laxmi et al., 2003; Antunes and Moita, 2010; Likhtik et al., 2014; Park et al., 2016; Xiao et al., 2018). When mice were exposed to pure tones of multiple frequencies, including the CS and UFs, for a long time, they showed a higher fear response to the CS (Figure 3F, right panel, Figure 3G, right panel, Figure 4F, right panel, Figure 4G, right panel). Previous discriminative fear conditioning experiments have found that most animals responded more strongly to the CS than to the CS- (Antoniadis and Mcdonald, 1999; Zelinski et al., 2010; Likhtik et al., 2014). The inhibition of the fear response by the pairing of the CS- and safety signals led to a lower fear response to the CS- in discriminative fear conditioning experiments (Likhtik et al., 2014). Nevertheless, the training process we performed was different from that of the discriminative fear conditioning experiments; we only paired pure tones of a single frequency (CS) with the US for fear training but did not pair the UFs with the US (Figure 1A). We speculate that there may be an emergent discriminative learning process resulting from additional multifrequency testing different from that observed from the discriminative fear conditioning experiments (Antoniadis and Mcdonald, 1999; Collins and Pare, 2000; Herry et al., 2008; Zelinski et al., 2010; Fenton et al., 2014). The discriminative learning was not an acquisition of fear memory during paired conditioning but was more like the process of comparing or connecting a retrieval of fear memory with different pure tones. It is worth noting that they have not yet reached the statistical difference between UF1 and UF2 in Figure 4F, left panel, Figure 4G, left panel, it is certainly correct that the further away you are from the CS frequency, the less likely an effect on freezing. We speculate that there is a rough frequency discrimination.

Furthermore, we also observed that the fear extinction of the CS was inhibited (Figures 3D,E, 4C,F, 5B), which may contribute to emergent discriminative learning. After training, the animals could not distinguish the CS when they were tested with pure tones of single frequencies in the extinction group (Figure 5D) but instead showed similar obvious extinctions of conditioned fear (Figures 5E,F). This finding indicates that the emergent discriminative learning was not due to the overconditioning of fear training but may be affected by the interaction between the CS and the UF. Previous literature supports our results in that the discrimination between the CS and the CS- was not determined by the response to the CS (Likhtik et al., 2014). In addition, we find that it is intermittent freezing during the testing. The freezing response after the CS presentation gradually decreased during the 30 s period (Supplementary Figure S2). The freezing response of mice in unit time (i.e., 1 s) is only 100 or 0%, and using the velocity of mice to quantify behavior will be more accurate. The smaller the velocity, the higher the freezing response.

We used muscimol to silence the mPFC (mainly corresponding to PL and IL) before multifrequency testing to determine whether the mPFC was related to the emergent discriminative learning (Figure 6). In the early stage of the testing, the fear response of the muscimol group was still high and not different from the control group (Figure 6C). This result confirmed and extended previous findings showing that the mPFC regulated fear responses by regulating the activity of the amygdala, which is involved in initial fear learning (Wilensky et al., 2006; Marek et al., 2018) and fear abatement (Lee et al., 2001; Walker et al., 2002; Burgos-Robles et al., 2007; Marek et al., 2018). The interaction between the mPFC and amygdala is also indispensable for fear expression and extinction (Laviolette et al., 2005; Likhtik et al., 2008; Sotres-Bayon and Quirk, 2010; Marek et al., 2018), while inactivation of the mPFC before testing and after fear training did not affect the expression of the fear response (Lee and Choi, 2012). In the latter stage of the testing, the discrimination between the CS and UF weakened or even disappeared in the muscimol group (Figure 6D, right panel). This result is consistent with the results of a previous study; when the mPFC was inactivated with muscimol before testing, the difference of fear responses between the CS− and CS+ disappeared (Lee and Choi, 2012). Moreover, there was obvious extinction of conditioned fear in the muscimol group (Figure 6F). This is in accordance with previous literature that indicates that inactivation of the mPFC did not affect the process of fear extinction (Burgos-Robles et al., 2007). Therefore, this study suggests that the specific recognition of the CS by mPFC neurons may be connected with the emergent discriminative learning elicited in the multifrequency testing after auditory fear conditioning.

Fear discrimination was associated with mPFC-to-BLA directionality, whereby the mPFC leads the BLA (Likhtik et al., 2014), and selective tuning of BLA firing to the theta input from the mPFC could serve as a mechanism through which the mPFC is able to signal safety and prevent overgeneralization (Spalding, 2018). Underactivation or reduced activity of the mPFC could lead to increased fear generalization (Giustino and Maren, 2015; Salm et al., 2015), and the disruption of N-methyl-D-aspartate receptor-dependent signaling or cAMP response element binding protein (CREB) and CREB binding protein (CBP) in the mPFC has been shown to lead to fear generalization (Vieira et al., 2014; Vieira et al., 2015). All of the investigations of non-human-animal cued fear conditioning were conducted at a recent time point, occurring within 1 day following acquisition (Spalding, 2018). Although damage to the ACC has been shown to reduce fear generalization in remote fear memories (Xu et al., 2012; Cullen et al., 2015; Einarsson et al., 2015; Spalding, 2018), damage to the IL, PL, and/or ACC was found to increase generalization in investigations of contextual fear in rodents conducted at a recent time point following acquisition (Antoniadis and Mcdonald, 2006; Xu et al., 2012; Sharpe and Killcross, 2014; Spalding, 2018). In the present study, we used cued fear conditioning but not contextual fear conditioning, tested mice at a recent (the following day) but not remote time point, and inactivated the PL and IL but not the ACC. Our experimental results were not inconsistent with previous studies.

In previous literature, labeled neurons in A1 were found projecting to mPFC after FlGo injection into mPFC in rats, and lidocaine injection in A1 cortices reduced SEPs and AEPs in mPFC (Martin-Cortecero and Nunez, 2016). We used lidocaine to silence ipsilateral A1 (mainly in the deep-layer) during the electrophysiological recordings, which involved pure tones and loose-patch recording (Figure 7). The effects of muscimol during blockade experiments can persist for up to 12–24 h, and lidocaine has a short induction time (within 5 min) and a short duration of action (up to 15–20 min), depending on the concentration, injected volume, and duration of injection (Martin and Ghez, 1999). Therefore, the effects of muscimol, but not lidocaine, can cover the entire behavioral testing period. Use of lidocaine in the electrophysiological experiments allows us to observe the recovery of responses, making sure the loose-patch state has not changed noticeably. Not only were the pure tone responses of mPFC neurons suppressed, but subsequent recovery of the responses was also observed (Figure 7B), which further validated the previous literature that indicating that the auditory information ascended to the mPFC mainly from A1 (Martin-Cortecero and Nunez, 2016). Under the same behavioral experimental procedure, we used muscimol to silence A1 on both sides before multifrequency testing (Figure 8). This procedure showed similar results with those from inactivating the mPFC, suggesting that pure tone information related to mPFC neurons participating in emergent discriminative learning elicited in the multifrequency testing after auditory fear conditioning may mainly come from A1. Previous studies have examined the relationship between the mPFC and A1 in auditory information processing. For example, the mPFC modulates the frequency receptive field plasticity (Yang et al., 2007) and the azimuth tuning plasticity of A1 (Gao et al., 2017), suggesting the possibility of consistency of auditory pathways in the mPFC and A1, which is in line with our experimental results. Another paper also supports the source of sound information in auditory fear discrimination. By combining local field potentials with multiunit recordings, the authors found that in animals that discriminate successfully between the CS and the UF, the activity of A1 and the PL became immediately and tightly synchronized in the slow gamma range (40–70 Hz) at the onset of the unrelated pure tone. This indicated that A1 and the PL established a functional connection and drove the behavioral choices of animals during auditory fear discrimination (Concina et al., 2018).

However, our current experimental results can’t strictly prove that mPFC hosts the circuits of discrimination learning. Pharmacological inactivation affects all cells in target area, not only sound responsive cells. Consequently, other less likely scenarios couldn’t be rigorously excluded. For example, it’s an alternative scenario that the discriminative learning in A1 (with cells showing an increased CS and unchanged non-CS responses), but that the result of the discrimination needs to be transmitted to mPFC for execution. In parallel, a fraction of mPFC cells could receive auditory inputs from A1, without it influencing the discriminative learning. In this scenario, the discrimination computation would occur in A1. In this study, we suggest that pure tone information related to mPFC neurons participating in emergent discriminative learning elicited in the multifrequency testing after auditory fear conditioning may mainly come from A1. While the pure tone response properties of mPFC neurons are different from those of A1 neurons, that is, most tone-responsive neurons in mPFC have high thresholds and broadband responses to pure tones. The effect of training on their pure tone response is not obvious in most individuals, but can be shown in general. It is also very puzzling that the best response in most mPFC neurons we recorded is to low frequencies. Moreover, the direct projection of mPFC and A1 is not strong (Supplementary Figure S5). We suggest that although the auditory information of mPFC originates from A1, it may have been integrated before arriving at mPFC. The other limitation of our experiment is that we didn’t follow changes in response properties in mPFC in the same animals before and after training. If we record the same animal with loose-patch recording and compare the changes before and after training, the data set that can be used for comparison will be smaller due to the unequal number of neurons. We also need to compare with the whole animals rather than individuals because of the poor comparison with a small number of neurons in the same animal. On the other hand, the tissues in the recording position of mouse are inevitably damaged to some extent after our electrophysiological recording. So it’s more difficult for us to record the same position of the mouse after training, and we are not sure how the damage will affect the result.

In future experiments, we could use opto-genetic targeting instead of pharmacological inactivation to specifically block the pathway of pure tones in discrimination learning. However, there may be several nuclei participating in this process between A1 and mPFC, and it requires more experimental design. We would learn and master extracellular single-unit recordings to replace loose-patch recordings in electrophysiological experiments. In that case, we would get a larger set of recorded cells in same animal and more convincing data to support our conclusion. Combining the above two methods may be helpful for further study of the more detailed path from A1 to mPFC in discrimination learning.

In some cases, especially for humans, fear discriminative learning is conducive to survival and development. In contrast, fear generalization reduces survival opportunities and leads to mental diseases, such as posttraumatic stress disorders and human phobias (Concina et al., 2018). However, in contrast, in other studies, authors have suggested that the generalization of fear is typically advantageous, as the same exact stimulus is rarely encountered again in the future (Spalding, 2018). Risk-related stimuli would invoke the same mechanisms that respond quickly and efficiently in the face of danger, and hence fear generalization with less specificity could be better for survival (Resnik et al., 2011; Resnik and Elliott, 2015). This study extends the related results of the mPFC in fear discriminative learning and acoustic responses. It shows that the relationship between the mPFC and A1 may be the key node in the process of fear discrimination and may be helpful to related psychiatric diseases such as phobias.

## Data Availability Statement

All datasets generated for this study are included in the article/Supplementary Material.

## Ethics Statement

All procedures were approved by the Animal Care and Use Committee of Southern Medical University, Guangzhou, China.

## Author Contributions

ZX and QL designed the research. XZ wrote the manuscript. XZ, XY, and RC performed the research and analyzed the data. All authors read and approved the manuscript.

## Conflict of Interest

The authors declare that the research was conducted in the absence of any commercial or financial relationships that could be construed as a potential conflict of interest.
