# Supplementary figures and images for: An Emergent Discriminative Learning Is Elicited During Multifrequency Testing
Source: Front Neurosci. 2019 Nov 21;13:1244. doi: 10.3389/fnins.2019.01244 (PMC6881306; doi:10.3389/fnins.2019.01244)

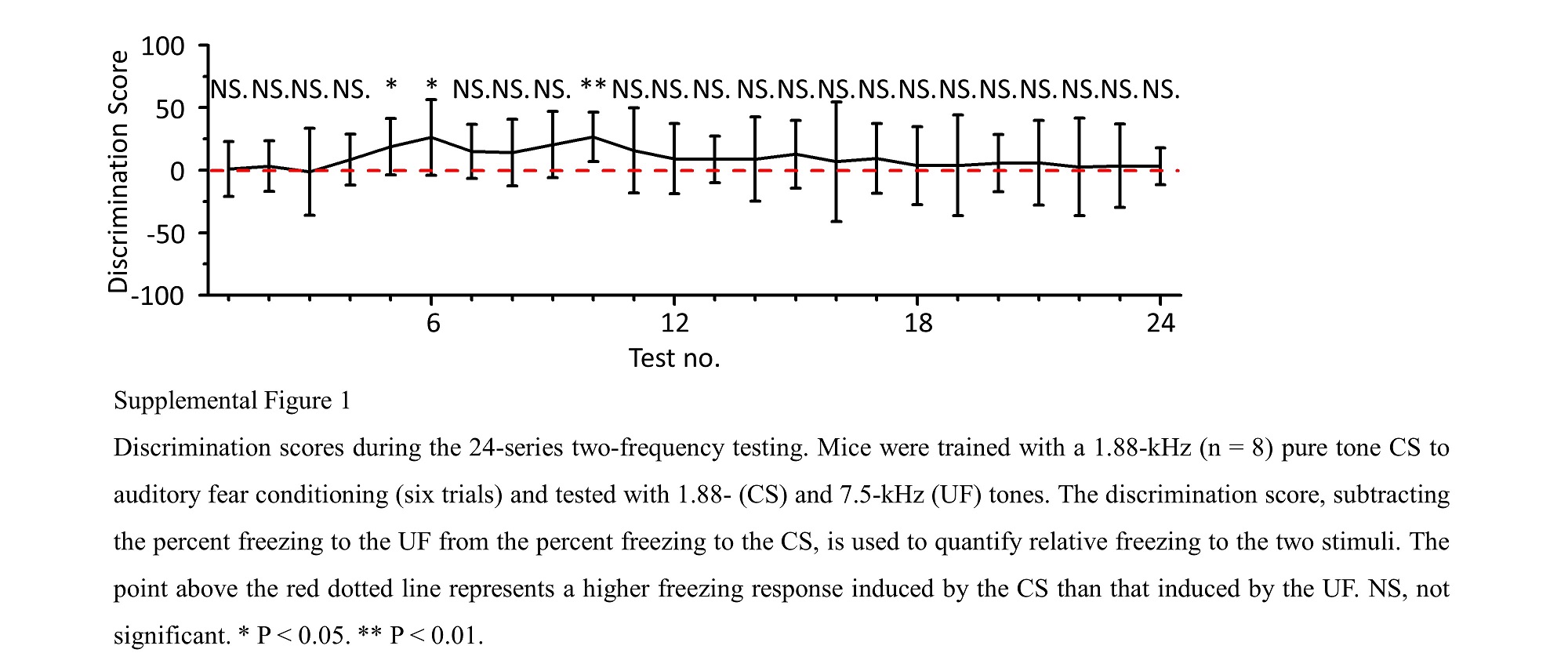

Supplement: Supplementary file 1 [file Image_1.JPEG]

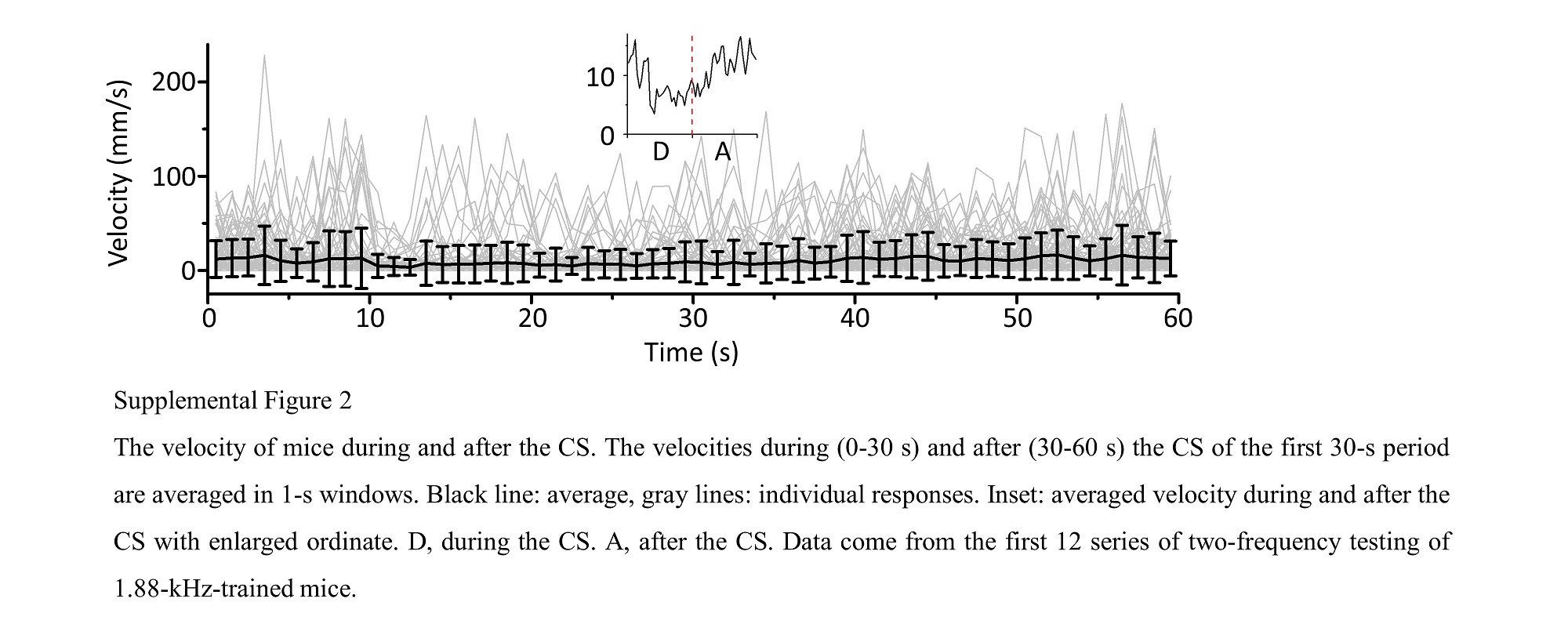

Supplement: Supplementary file 2 [file Image_2.JPEG]

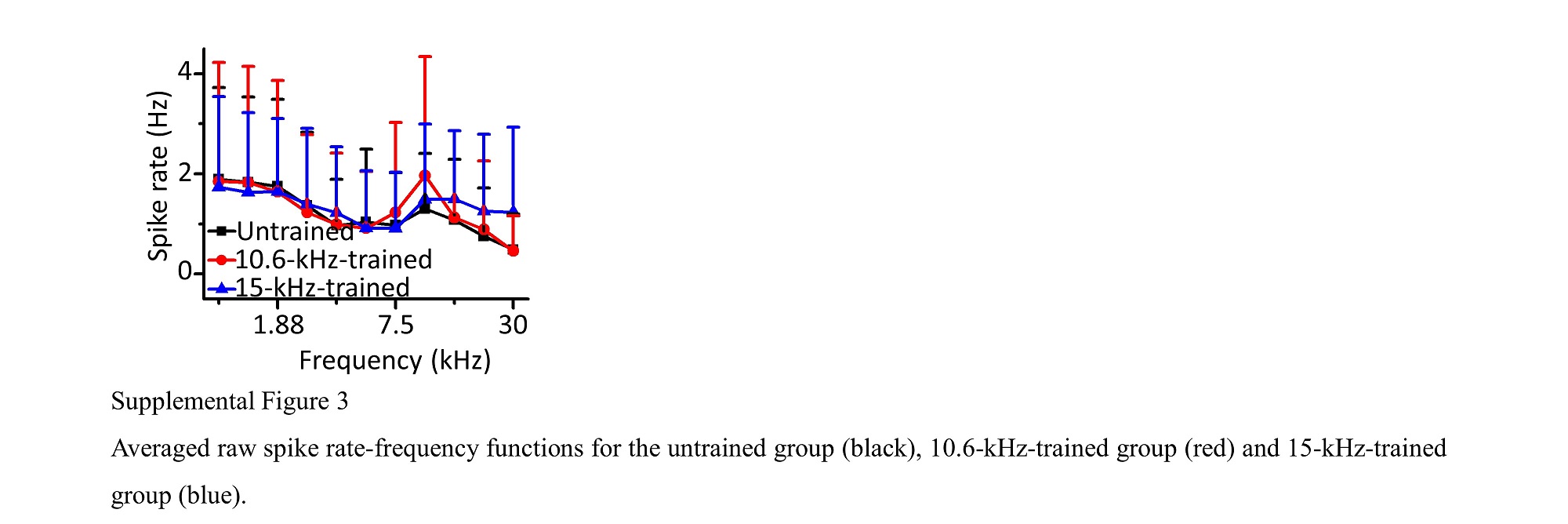

Supplement: Supplementary file 3 [file Image_3.JPEG]

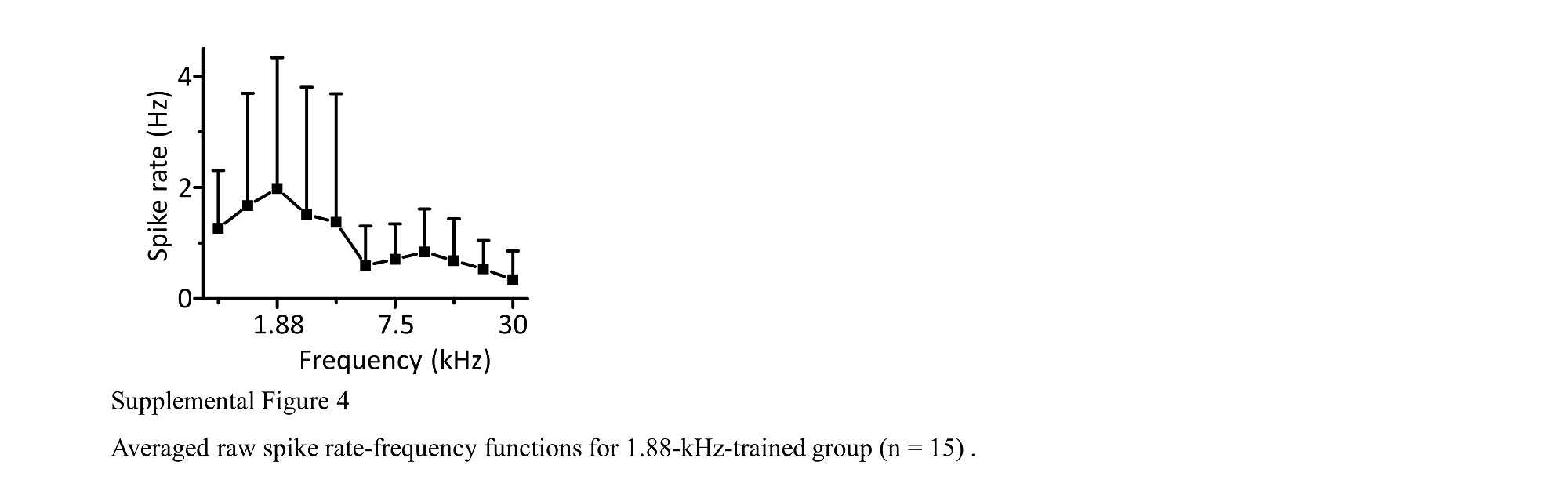

Supplement: Supplementary file 4 [file Image_4.JPEG]

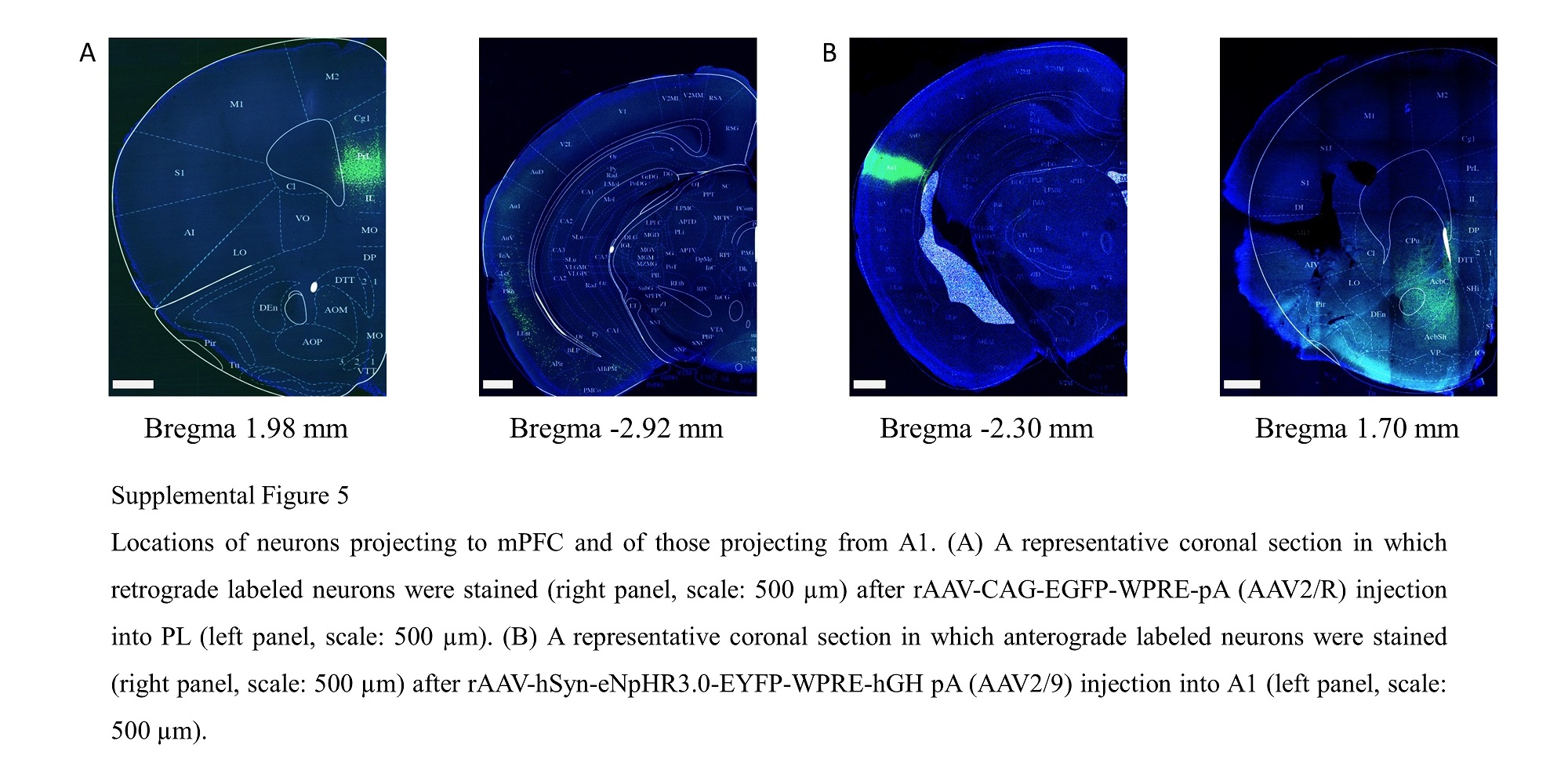

Supplement: Supplementary file 5 [file Image_5.JPEG]

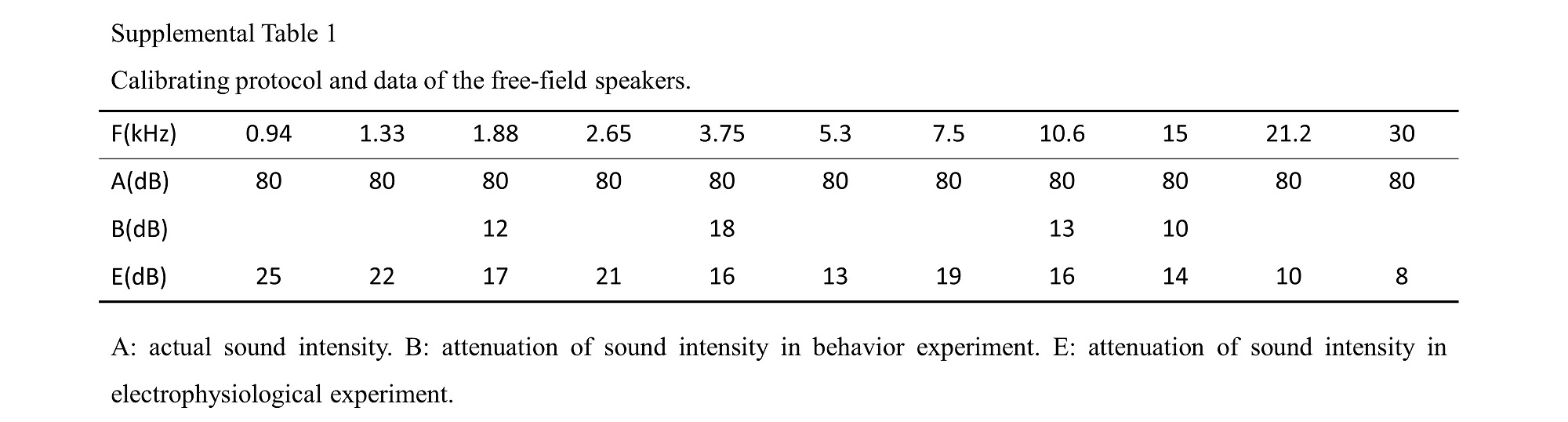

Supplement: Supplementary file 6 [file Image_6.JPEG]

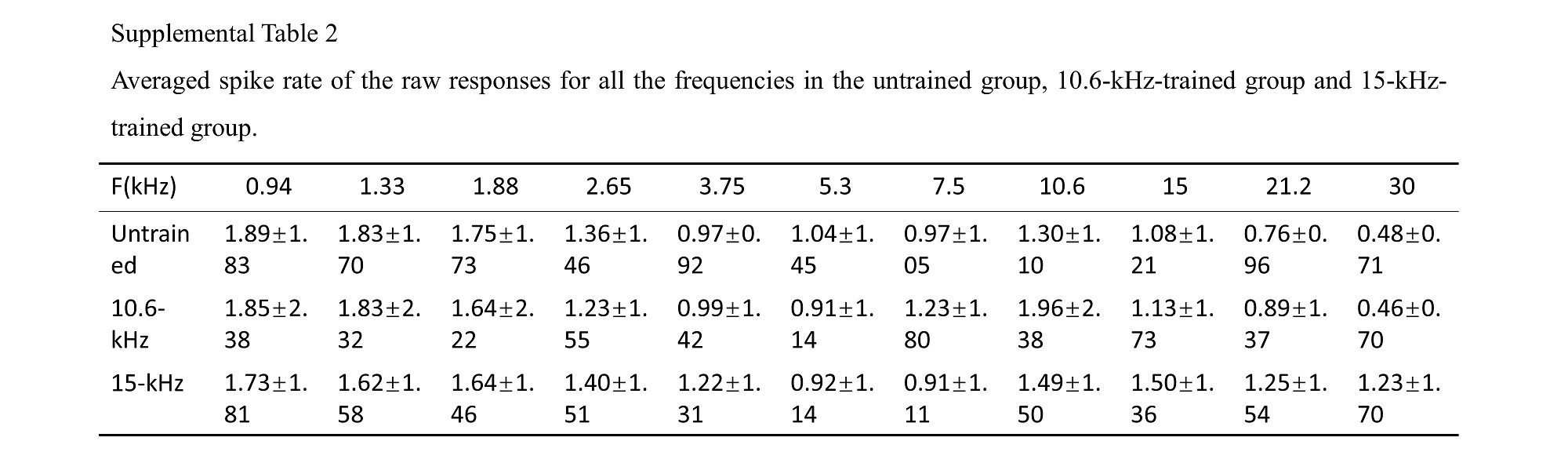

Supplement: Supplementary file 7 [file Image_7.JPEG]

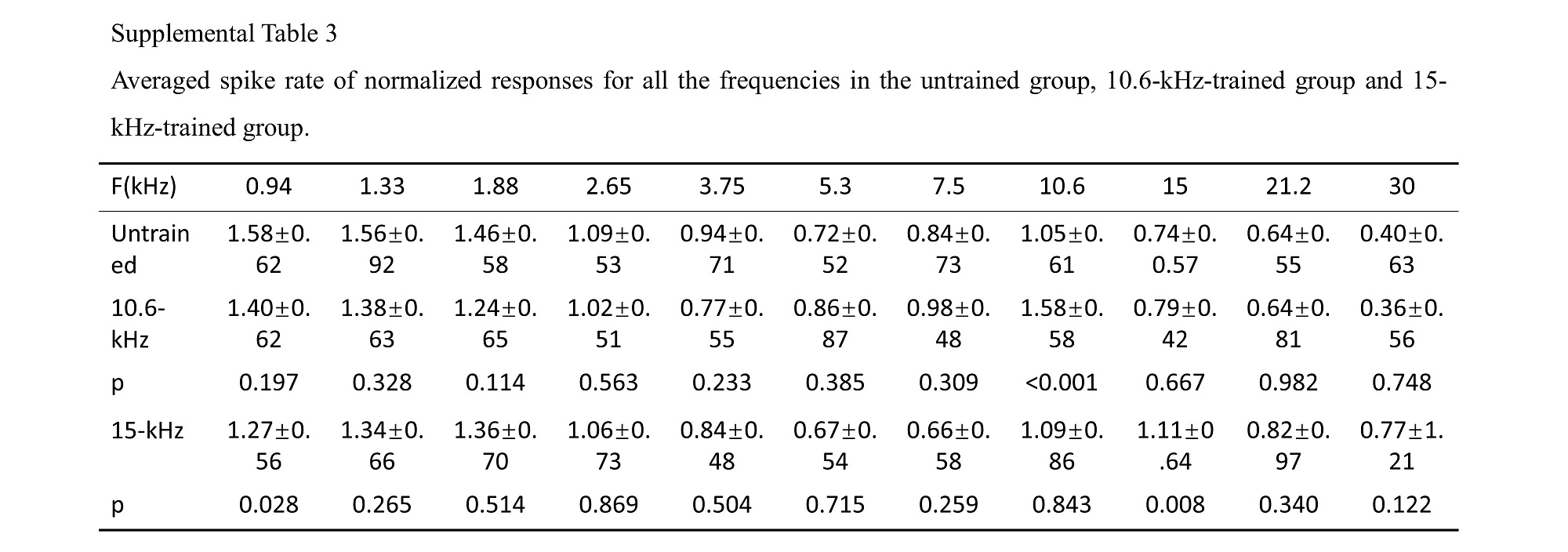

Supplement: Supplementary file 8 [file Image_8.JPEG]
